# Patellar resurfacing and kneeling ability after total knee arthroplasty: a systematic review

**DOI:** 10.1186/s42836-023-00184-5

**Published:** 2023-06-03

**Authors:** Owais A. Shah, Christopher Spence, Deiary Kader, Nick D. Clement, Vipin Asopa, David H. Sochart

**Affiliations:** 1 South West London Elective Orthopaedic Centre, Epsom, KT18 7EG UK; 2 Edinburgh Orthopaedics, Royal Infirmary of Edinburgh, Edinburgh, EH16 4SA UK

**Keywords:** Arthroplasty, Knee, Total knee arthroplasty, Patellar resurfacing, Kneeling, Kneel

## Abstract

**Background:**

Difficulty kneeling following total knee arthroplasty (TKA) remains highly prevalent, and has cultural, social, and occupational implications. With no clear evidence of superiority, whether or not to resurface the patella remains debatable. This systematic review examined whether resurfacing the patella (PR) or not (NPR) influences kneeling ability following TKA.

**Methods:**

This systematic review was conducted by following PRISMA guidelines. Three electronic databases were searched utilizing a search strategy developed with the aid of a department librarian. Study quality was assessed using MINROS criteria. Article screening, methodological quality assessment and data extraction were performed by two independent authors, and a third senior author was consulted if consensus was not reached.

**Results:**

A total of 459 records were identified, with eight studies included in the final analysis, and all deemed to be level III evidence. The average MINORS score was 16.5 for comparative studies and 10.5 for non-comparative studies. The total number of patients was 24,342, with a mean age of 67.6 years. Kneeling ability was predominantly measured as a patient-reported outcome measure (PROM), with two studies also including an objective assessment. Two studies demonstrated a statistically significant link between PR and kneeling, with one demonstrating improved kneeling ability with PR and the other reporting the opposite. Other potential factors associated with kneeling included gender, postoperative flexion, and body mass index (BMI). Re-operation rates were significantly higher in the NPR cohort whereas PR cohorts had higher Feller scores, patient-reported limp and patellar apprehension.

**Conclusion:**

Despite its importance to patients, kneeling remains not only under-reported but also ill-defined in the literature, with no clear consensus regarding the optimum outcome assessment tool. Conflicting evidence remains as to whether PR influences kneeling ability, and to clarify the situation, large prospective randomized studies are required.

## Background

Despite COVID-19 disruptions, 226,350 primary total knee arthroplasties (TKAs) were carried out within England and Wales between January 2018 to December 2020 as per National Joint Registry (NJR) data and it is estimated that, by the year 2030, about 3.48 million TKAs will be performed annually [1, 2]. TKA remains an effective management option for end-stage osteoarthritis (OA) of the knee, and has demonstrated safe and reproducible long-term results with regards to improvement in pain and quality of life. TKA has been shown to significantly improve physiological knee alignment, patient-reported outcome measures (PROMs), functional scores and to confer postural benefits, such as centre of gravity correction and normalization of both pressure and body-weight displacement through the operated limb in the early postoperative period [3]. Regardless of these benefits, approximately one in five patients remain dissatisfied with the outcome of their surgery [4].

Cohort studies revealed that post-TKA satisfaction ranged from 81%–89%, with a large UK cohort study exhibiting that 18.6% of 1217 consecutive patients were either unsure of or dissatisfied with their results one-year post-TKA [4–6]. Many factors have been implicated in the post-TKA dissatisfaction, including age, gender, mental health scores, personality traits, preoperative morbidity and pain scores [4]. Rotational alignment of TKA prosthetic components and maintaining correct mechanical axes are related to optimal functional recovery following surgery [7]. A retrospective study defined a new patellar angle to be used in the early diagnosis of prosthetic rotational malalignment, which has been implicated in the prediction of the incidence of anterior knee pain after surgery [7]. Nevertheless, the strongest predictor for dissatisfaction remains unmet patient expectations with regards to functional and symptomatic improvement after surgery [5]. One such outcome measure, which, despite being rated highly important by patients, fails to meet preoperative expectations of improvement, is the ability to kneel [8, 9].

Prost defined kneeling in 1974 as a postural position in which at least one knee is in contact with the ground while body-weight is supported predominantly through the knees [10]. Different positional patterns can fulfill this definition, including single-leg kneeling, upright kneeling, high flexion kneeling and praying position kneeling. Activities of daily living as well as many leisure activities, such as cleaning, decorating, gardening, sports, and exercise, are impacted by an inability to kneel, with patients often needing adjustments or relying on support from friends and family to compensate, negatively impacting their emotional state, social independence, and well-being [11]. Kneeling is also an important function for dining and social participation in east Asian cultures [12] and of religious practice among both the Christian and Islamic faith, with followers of the latter requiring high flexion kneeling for daily prayer [13, 14]. Many occupations require kneeling, such as plumbing, cleaning, roofing and floor laying [15]. Despite its importance, kneeling remains the activity least improved following surgery, with one-third of patients not returning to work following TKA [16].

Kneeling ability is consistently the poorest of the patient-reported outcome measures (PROMs) following TKA and remains prevalent in both the short and longer terms, with one study revealing that 67% of patients reported much difficulty or found it impossible to kneel five years after TKA [17]. Kneeling ability is usually assessed as a self-reported outcome measure. Its assessment commonly uses question seven of the Oxford Knee Score (OKS), which rates it on a five-point ordinal scale, ranging from 4 ("yes, easily") to 0 ("no, impossible"). The OKS has been adopted by the UK government and the NJR as a validated tool to assess outcomes [4].

Associations between intraoperative variables and postoperative kneeling ability have been investigated with contradictory results. One such variable is whether or not to resurface the patella during TKA, which remains largely at the discretion of the operating surgeon [11, 18]. This review investigated whether PR or NPR influences the ability to kneel following TKA.

## Methods

This systematic review was conducted by following Preferred Reporting Items for Systematic Reviews and Meta-Analyses (PRISMA) guidelines [19] and was registered with PROSPERO (International Prospective Register of Systematic Reviews), (ID = CRD42022306341) [20].

### Search strategy and eligibility criteria

A systematic search strategy and syntax were developed with the aid of the department librarian. A combination of Medical Subject Heading (MeSH) terms and keywords were incorporated to electronically search EMBASE and Medline libraries using Healthcare Database Advanced Search (HDAS) and the native PubMed database from inception to October 2021 (Table 1). An additional grey literature search was performed using Open Grey, and the reference lists of included studies were reviewed to identify articles missed by the original search strategy.

**Table 1:** Strategies used in pubmed search

Search number	Query	Results
6	#3 and #4 and #5	182
5	(“resurfac*” or “patella* resurfac*” or “patellofemoral resurfac*” or “patello-femoral resurfac*” or “patellofemoral joint resurfac* “ or “patellofemoral joint replacement” or “patello-femoral joint resurfac*” or “patello-femoral joint replacement” or “patello-femoral arthroplasty” or “patellofemoral arthroplasty”)	7,963
4	(“native patell*” or “non-resurfac*” or “nonresurfac*” or “non resurfac*” or “non-resurfac*” or “nonresurfac*” or “patella* nonresurfac*” or “patella* non-resurfac*” or “patella* non resurfac*” or “patellofemoral nonresurfac*” or “patellofemoral non-resurfac*” or “patellofemoral non resurfac*” or “patellofemoral joint nonresurfac*” or “patellofemoral joint non-resurfac*” or “patellofemoral joint non resurfac*”)	244
3	#1 or #2	43,895
2	“Arthroplasty, Replacement, Knee” [MeSH]	27,414
1	(“total knee replacement arthroplasty” or “total knee replacement” or “total knee prosthesis” or “total knee prostheses” or “total knee joint replacement” or “total knee arthroplasty” or “total arthroplasty” or “knee replacement” or “total knee replacement*” or TKR or “total knee arthroplast*” or TKA or “total knee prosthes*” or “knee prosthes*” OR “knee arthroplast*”)	43,895

(* = Highlighting capture of additional possible variations on search term incorporated within search strategy)

Inclusion criteria were (1) Studies involving patients undergoing a TKA, with PR or NPR, with kneeling outcomes reported, (2) Papers published in English with full text available, (3) Peer reviewed clinical studies. Exclusion criteria included (1) Studies presenting non-original data, case reports, review studies, conference abstracts, editorials, opinion papers and letters to the editor. (2) Mixed cohort of patellar resurfaced (PR)/non-resurfaced (NPR) TKA patients with no direct comparative analysis of kneeling outcomes.

### Article screening

Duplicates were removed from the list of papers identified. Titles and abstracts were screened by two independent reviewers (OAS, CS), who then screened the full-text papers of relevant studies. The final study selection was completed by two independent reviewers as per pre-defined inclusion/exclusion criteria. Any inconsistencies were discussed with a third, senior reviewer (DHS) available for consultation if consensus was not achieved.

### Level of evidence and methodological quality

The methodological quality of the studies was scored by two independent reviewers (OAS, CS) using the Methodological Index for Non-Randomized Studies (MINORS) tool [21]. The level of evidence of each study was reported by two reviewers as per the Oxford Centre for Evidence-Based Medicine (OCEBM) Levels of Evidence [22]. Critical appraisal of included studies was performed by two independent reviewers (OAS, CS) with a third, senior reviewer (DHS) present to resolve potential failure to reach a consensus.

### Data extraction

Two independent authors (OAS, CS) extracted data from the included studies, comprising study characteristics, patient demographics, implant characteristics, surgical details, details of kneeling outcomes compared between PR and NPR, details of variables associated with kneeling outcomes other than PR, variables other than kneeling compared between PR and NPR cohorts, study conclusions and limitations.

Based on the heterogeneity in study data particularly in the tools utilized to assess kneeling ability, it was decided a quantitative meta-analysis was not feasible.

## Results

### Search results

**Fig. 1:**
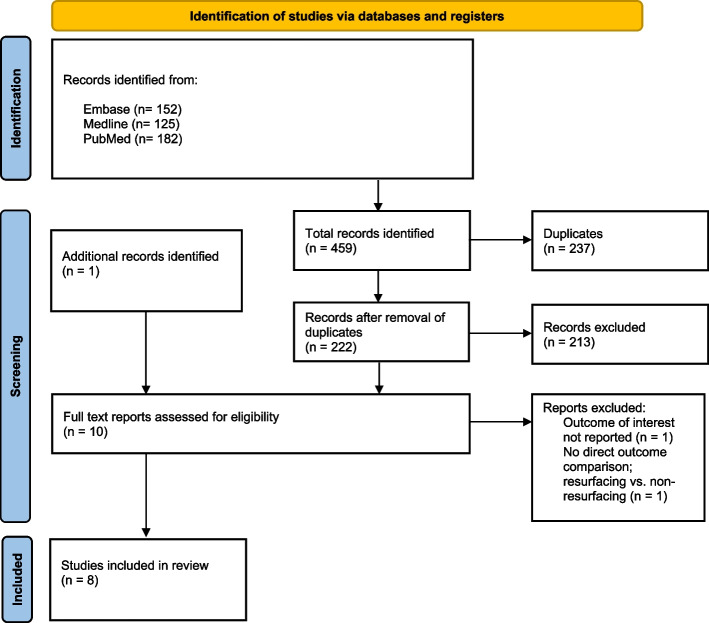
PRISMA 2020 flow diagram for new systematic reviews which included searches of databases and registers only

The electronic search of the EMBASE and Medline libraries via HDAS and the native PubMed database identified 459 articles. After removing duplicates and irrelevant records based on titles and abstracts, nine articles were eligible for full-text screening. One additional record was identified by reviewing reference list of a relevant review article. No articles were identified by a search of grey literature. Full texts for a total of ten articles were screened with two records excluded as per inclusion/exclusion criteria, leaving eight records included in the final qualitative analysis of the review (Fig. 1).

### Risk of bias and quality assessment

**Fig. 2:**
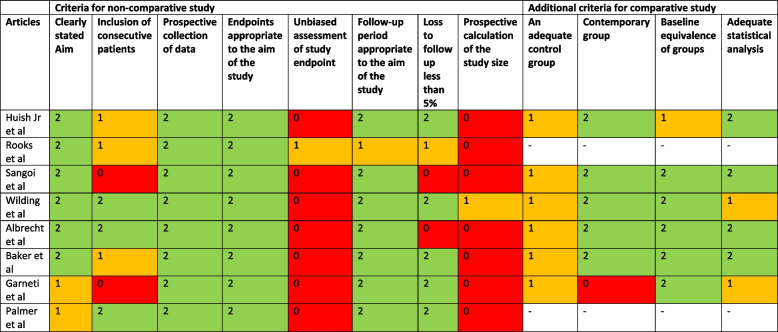
Risk of bias assessment using MINORS criteria

All eight studies included were of retrospective cohort design with a level of evidence of III [23–30]. Average MINORS score was 16.5 (SD = 1.98) for comparative studies [23, 25–29] and 10.5 (SD = 0.5) for non-comparative studies [24, 30]. Risk of bias assessed using MINORS criteria is presented in Fig. 2. Table 2 shows the level of evidence and quality assessment of each study included.

**Table 2:** Year, study design, country of origin, level of evidence and MINORS score of included studies

Author	Year	Study design	Country	LE	MINORS score
Huish Jr et al	2020	Retrospective Cohort Study	USA	III	17/24
Rooks et al	2020	Retrospective Cohort Study	Canada	III	10/16
Sangoi et al	2020	Retrospective Cohort Study	UK	III	15/24
Wilding et al	2019	Retrospective Cohort Study	UK	III	19/24
Albrecht et al	2016	Retrospective Cohort Study	Switzerland	III	17/24
Baker et al	2014	Retrospective Cohort Study (NJR Study)	UK	III	18/24
Garneti et al	2008	Retrospective Cohort Study	UK	III	13/24
Palmer et al	2002	Retrospective Cohort Study	Australia	III	11/16

*NJR* National Joint Registry, *LE* Level of evidence, *MINORS* Methodological index for non-randomized studies

### Study and cohort characteristics

All eight studies were published between 2002 and 2020. Four were UK-based, with one each from the USA, Canada, Australia, and Switzerland (Table 2). The total number of patients involved in the studies was 24,342, with a mean age of 67.6 years (range 63.4–71.6) of the studies that reported this. The total number of patients was 8,625 in the PR cohort and was 15,689 in NPR cohort, with one study not reporting the number of patients by whether they had PR or NPR. Cohort characteristics are detailed in Table 3.

**Table 3:** Summary of study characteristics including description of primary outcome (kneeling) measurement

**Study**	**Mean follow up (months)**	**Mean age (years)**	**Number of patients**	**Number of knees**	**Resurfacing**					**Non-resurfacing**					**PROM —kneeling**	**Clinical assessment of kneeling**
					**Number of patients**	**Mean age (years)**	**Male**	**Female**	**Follow up (months)**	**Number of patients**	**Mean age (years)**	**Male**	**Female**	**Follow up (months)**		
Huish Jr et al (2020) [23]	NR	66.5	84	NR	56	67.8	20	36	Mean = 48	28	63.8	6	22	Mean = 57	Questionnaire^a^	No
Rooks et al (2020) [24]	NR	67.8	420	NR	242	NR	NR	NR	NR	178	NR	NR	NR	NR	Questionnaire^b^	No
Sangoi et al (2020) [25]	NR	NR	104	NR	62	65.2	NR	NR	Mean = 54.9	42	65.8	NR	NR	Mean = 74.5	OKS (Q5 + 7 + 12)	No
Wilding et al (2019) [26]	39.6	71.6	79	100	NR	NR	NR	NR	NR	NR	NR	NR	NR	NR	Questionnaire^c^	Yes^f^
Albrecht et al (2016) [27]	65.6	66.3	66	71	51	67	26	35	NR	20	64.6	12	8	NR	KOOS (SP5)	No
Baker et al (2014) [28]	NR	NR	23,393	NR	8103	69.6	3180	4923	Median = 6.5	15,290	69.5	6990	8300	Median = 6.5	OKS (Q7)	No
Garneti et al (2008) [29]	NR	NR	121	142	76	71	34	42	Mean = 33	66	74	30	36	Mean = 18	Questionnaire^d^	No
Palmer et al (2002) [30]	30	66	75	100	35	NR	NR	NR	NR	65	NR	NR	NR	NR	Questionnaire^e^	Yes^g^

*PROM* Patient reported outcome measure, *NR* Not reported, *Q* Question, *OKS* Oxford Knee Score, *KOOS* Knee Injury and Osteoarthritis Outcome Score, *CKRS* Clinical Knee Rating System, *VAS* Visual Analog Scale

^a^Subjective ability to kneel with ability to kneel defined as patient reporting they could kneel easily, with little or moderate difficulty, and inability to kneel was defined as patients reporting moderate or extreme difficulty kneeling

^b^Patients contacted by telephone to participate in a structured questionnaire, including questions regarding ability to kneel (yes/no) and follow-up (if no-, why not)

^c^Patients asked via questionnaire if able to kneel before and after TKA

^d^Patients self-reporting their ability to kneel

^e^Patients were asked about ability to kneel and to record level of pain 0–10. Two groups emerged: those able to kneel without pain or with only mild pain (score 0–4) and those unable to kneel because of pain in the knee (score 5–10)

^f^Patients were asked to kneel on padded examination couch and then onto a pillow on the floor. Degree of flexion achievable was recorded

^g^Patients were asked to demonstrate kneeling on a hard surface

### Assessment of kneeling ability

All eight studies recorded kneeling as a PROM. One study used question SP5 from the Knee Injury and Osteoarthritis Outcome Score (KOOS) [27]. One study used question 7 of the OKS [28], with another using cumulative sum of questions 5,7 and 12 of the OKS to assess kneeling [25]. The remaining five studies used non-validated questionnaires [23, 24, 26, 29, 30]. Two studies utilized an additional objective clinical assessment to report kneeling outcomes [26, 30] and the details regarding objective and subjective measured kneeling outcomes are presented in Table 3.

### Surgical techniques and implant characteristics

Three studies used a cruciate-retaining (CR) implant preserving the posterior cruciate ligament (PCL) [23, 29, 30], one used a medial rotation design sacrificing the PCL [25] and one used CR implants in 56% of cases and a cruciate sacrificing (CS) implant in 44% [24]. Three studies did not report on the implant used [26–28] (Table 4).

**Table 4:** Surgical techniques including implants used

**Study**	**Implant**	**PS vs. CR vs. others**	**Approach**	**PCL sacrificed vs. retained**	**Tourniquet used**	**Drains used**
Huish Jr et al. [23]	U2 Knee	CR	Medial parapatellar or subvastus	Retained	NR	NR
Rooks et al [24]	Zimmer NexGen Total Knee	CR (56%) and CS (44%)	NR	NR	NR	NR
Sangoi et al. [25]	MRK	Other	Medial Parapatellar	Sacrificed	Yes	No
Wilding et al. [26]	AGC/AGC PS, Vanguard/Vanguard PS, PFC PS, RHK	NR	NR	NR	NR	NR
Albrecht et al. [27]	E.Motion UC prosthesis with rotating inlay	NR	NR	NR	NR	NR
Baker et al. [28]	NR	NR	NR	NR	NR	NR
Garneti et al. [29]	Scorpio TKA	CR	NR	Retained	NR	NR
Palmer et al. [30]	NR	CR	Medial parapatellar	Retained	NR	NR

*NR* Not reported, *TKA* Total knee arthroplasty, *TKR* Total knee replacement, *PS* Posterior stabilizing, *CR* Cruciate retaining, *PCL* Posterior cruciate ligament, *MRK* Medial knee rotation, *PFC* Press fit condylar, *RHK* Rotating hinge knee

In two studies, a medial parapatellar approach [25, 30] was used, and in one, either a medial parapatellar or a subvastus approach [23] was used, with the remaining studies not reporting the approach. None of the eight studies reported the specifics of the skin incision or whether mobile or fixed-bearing prostheses were used. One study documented that drains had not been used [25].

### Kneeling ability following TKA

Two studies reported a statistically significant link between PR and kneeling following TKA [23, 26]. Huish et al. reported a significantly greater (64% vs. 39%) ability to kneel with NPR at 2.5 years follow-up, with the ability to kneel defined as patients reporting that they could kneel easily, with little or moderate difficulty [23]. In contrast, Wilding et al. employed an objective assessment of kneeling ability, with inability defined as being unable to kneel on either a soft couch or hard floor due to discomfort or pain. They found that 78.6% of patients with PR were able to kneel compared to 45.6% of NPR patients, with the difference being statistically significant [26] (Tables 5, 6).

**Table 5:** Studies demonstrating a statistically significant difference in kneeling outcomes compared between patellar resurfacing vs. non-resurfacing

**Study**	**Year**	**Statistical analysis comparing kneeling in resurfaced vs. non-resurfaced TKAs**	**Study conclusions regarding kneeling (Resurfacing vs. non-resurfacing)**
Huish Jr et al. [23]	2020	Favors Non-resurfacing	1. Not resurfacing the patella, combined with lateral facetectomy may give patients a better chance to kneel
			2. Without evidence clearly demonstrating whether patellar resurfacing or non-resurfacing is superior, the surgeon should give consideration to not resurfacing the patella in patients with minimal patellofemoral arthritis that may have desire/need to kneel following surgery
			3. Female patients showed an increased ability to kneel, hence leaving their native patellar surface may aid in kneeling activities after surgery
Wilding et al. [26]	2019	Favors Resurfacing	1. Patella resurfacing significantly improves patients’ chances of being able to kneel post TKA
			2. This benefit is independent of whether PCL is retained or sacrificed

**Table 6:** Comparison of the ability to kneel among patients with patellar resurfacing vs. non-resurfacing

**Study**	**Year**	**Assessment of kneeling**	**Ability to kneel**		**Significance**	***P* = value**
			**Resurfaced (PR)**	**Non-resurfaced (NPR)**		
Huish Jr et al. [23]	2020	Self-reported	56 (39%)	28 (64%)	Significant	*P* = 0.04* PR 95% CI (0.26–0.52) NPR 95% CI (0.47–0.82)
Rooks et al [24]	2020	Self-reported	Can kneel = 69 (29%)	Can kneel = 54 (30%)	Not significant	NR
			Limited kneeling = 56 (23%)	Limited kneeling = 35 (20%)	Not significant	NR
			Cannot kneel = 117 (48%)	Cannot kneel = 89 (50%)	Not significant	NR
Sangoi et al. [25]	2020	Self-reported-OKS (Q5 + Q7 + Q12)	Median (range)	Median (range)	NR	NR
			Preop = 3 (0–12)	Preop = 3 (1–11)		
			Postop = 8 (2–12)	Postop = 6.5 (3–11)		
			Change = Significant (*P* = 0.039)			
Wilding et al. [26]	2019	Clinical assessment	Able to kneel = 33 (79%)	Able to kneel = 26 (46%)	Significant	*P* < 0.01* PR 95% CI (0.66–0.91) NPR 95% CI (0.33–0.59)
			Unable to kneel = 9 (21%)	Unable to kneel = 31 (54%)		
			Implant design–PCL Sacrificed	Implant design–PCL Sacrificed	Not significant	*P* = 0.34
			Able to kneel = 17 (68%)	Able to kneel = 10 (42%)		
			Unable to kneel = 8 (32%)	Unable to kneel = 14 (58%)		
			Implant design–PCL Retained	Implant design–PCL Retained	Not significant	*P* = 0.06
			Able to kneel = 16 (94%)	Able to kneel = 16 (48%)		
			Unable to kneel = 1 (6%)	Unable to kneel = 17 (52%)		
Albrecht et al. [27]	2016	Self-reported—KOOS (SP5)	Mean (SD)	Mean (SD)	Not significant	*P* = 0.72
			2.22 (1.60)	1.45 (1.54)		
Baker et al. [28]	2014	Self-reported—OKS (Q7)	Mean (95% CI)	Mean (95% CI)	Significant^a^	*P* < 0.01*
			Preop = 0.75 (0.73 to 0.77)	Preop = 0.82 (0.81 to 0.83)		
			Postop = 1.36 (1.33 to 1.39)	Postop = 1.41 (1.39 to 1.43)	Significant^a^	*P* < 0.01*
			Change = 0.61 (0.58 to 0.64)	Change = 0.59 (0.57–0.61)	Not significant	*P* = 0.29
Garneti et al. [29]	2008	Self-reported	Able to kneel = 21 (31%)	Able to kneel = 17 (31%)	Not significant	*P* = 0.09
Palmer et al. [30]	2002	Clinical assessment	Able to kneel = 26 (84%)	Able to kneel = 38 (67%)	Not significant	*P* = 0.1
			Unable to kneel = 5 (16%)	Unable to kneel = 19 (33%)		

^*^ = *P* < 0.05, *NR* Not reported, *CI* confidence interval, *PR* patella resurfacing, *NPR* Non-patella resurfacing

^a^Changes in OKS kneeling scores (Q7) between resurfaced vs. non-resurfaced groups were not significant once adjusted for differences in age, gender, ASA grade, surgical indications, preoperative general health, preoperative history of depression and relevant preoperative PROM score in multivariate analysis

Sangoi et al. used the summation of OKS questions 5, 7 and 12 to assess self-reported kneeling ability, although only question 7 is specifically about kneeling. They showed that the PR cohort reported greater improvements compared to the NPR group, and the improvement postoperatively was statistically significant for the PR group but not the NPR group [25]. Baker et al. reported a statistically significant difference between pre- and postoperative self-reported kneeling scores measured using question 7 of the OKS between the PR and the NPR cohorts, but this was not significant once adjusted for multivariate analysis [28]. None of the other studies reported a statistically significant difference in kneeling outcomes between PR and NPR patients (Table 6).

Huish Jr et al. reported that kneeling ability was significantly higher in female patients [23], but conversely, Rooks et al. found that males were significantly more likely to self-report being able to kneel [24]. Although age and type of implant did not impact kneeling ability in their study, they also reported an inverse relationship between body mass index (BMI) and kneeling ability, with patients having a BMI greater than 33 being significantly less likely to be able to kneel [24]. Palmer et al. reported no significant link between postoperative flexion or Knee Society Score (KSS) and kneeling ability [30], although Wilding et al. reported a significant link between postoperative flexion and kneeling ability, with knee flexion to 100° or greater demonstrating a higher ability to kneel [26]. Age, status of PCL, or type of surgery (revision vs. primary) did not influence kneeling ability and variables other than PR and their impact on kneeling are presented in Table 7.

**Table 7:** Variables other than ‘resurfacing vs. non-resurfacing’, associated with kneeling post TKA

**Study**	**Year**	**Variables**	**Significance**	***P* = value**	**Conclusions**
Huish Jr et al. [23]	2020	Gender	Significant	0.02*	Kneeling ability higher in females
Rooks et al [24]	2020	Gender	Significant	< 0.01*	Kneeling ability higher in males
		BMI	Significant	NR	Patients with BMI > 33 was less likely to be able to kneel
		Age	Not significant	NR	
		Type of Implant	Not significant	NR	
Wilding et al. [26]	2019	Post-operative flexion	Significant	0.01*	Knees that were able to flex ≥ 100° were more likely to be able to kneel
		Age	Not significant	0.54	
		PCL retaining vs sacrificing prostheses	Not significant	0.54	
		Revision vs Primary TKA	Not significant	0.41	
Palmer et al. [30]	2002	Post-operative flexion	Not significant	0.60	
		KSS	Not significant	0.60	

^*^ = *P* < 0.05, *BMI* Body mass index, *KSS* Knee society score

### Patellar resurfacing vs. non-resurfacing

Outcomes other than kneeling compared between PR and NPR are presented in Table 8. Huish Jr et al. reported no significant difference in OKS scores between PR and NPR patients [23]. This was also reported by Sangoi et al. and Baker et al., who found no significant difference in either pre- or postoperative OKS scores when comparing the PR and NPR groups [25, 28]. Garneti et al. found no significant difference in the PR and NPR groups when comparing Euroqol scores and KSS scores [29], with Albrecht et al. also reporting no difference in KSS scores [27]. Sangoi et al. reported higher Feller scores in the PR group [25]. Garneti et al. reported a significantly higher revision rate in the NPR group but higher patient-reported limp and patellar apprehension in the PR group [29]. Albrecht et al. found no significant differences in ROM or revision rates [27].

**Table 8:** Outcomes other than ‘kneeling’ compared in patients with patellar resurfacing against those with non-resurfacing

**Study**	**Year**	**Outcome measured**	**Resurfaced**	**Non-resurfaced**	**Significance**	*P-***Value**
Huish Jr et al. [23]	2020	OKS	39	38	Not significant	NR
		VAS	2.5	3	Not significant	NR
		Re-operation rate	2 (4%)	2 (7%)	Not significant	NR
Rooks et al [24]	2020	Patient reported satisfaction	Very satisfied = 190 (79%)	Very satisfied = 129 (71%)	Not significant	NR
			Partially satisfied = 44 (18%)	Partially satisfied = 40 (22%)	Not significant	NR
			Not satisfied = 8 (3%)	Not satisfied = 9 (5%)	Not significant	NR
			Have TKA again = 211 (87%)	Have TKA again = 149 (84%)	Not significant	NR
			Do not have TKA again = 31 (13%)	Do not have TKA again = 29 (16%)	Not significant	NR
Sangoi et al. [25]	2020	OKS	Median (range)	Median (range)	Not significant	NR
			Preop = 14 (1–44)	Preop = 15 (4–42)		
			Postop = 37 (9–48)	Postop = 36 (1–47)	Not significant	NR
		Baldini Score	Median (range)	Median (range)	Not significant	*P* = 0.07
			100 (30–100)	90 (5–100)		
		Feller Score	Median (range)	Median (range)	Significant	*P* = 0.04 *
			28 (10–30)	25 (12–30)		
Albrecht et al. [27]	2016	Mechanical tibiofemoral angle	Mean (SD) = 0.22 (2.42)	Mean (SD) = 0.55 (3.22°)	Not significant	*P* = 0.64
		ROM	Mean (SD) = 115.6° (16.0°)	Mean (SD) = 117.9° (11.0°)	Not significant	*P* = 0.59
		KSS	Total score Mean (SD) = 173.8 (20.7)	Total score Mean (SD) = 176.7 (19.5)	Not significant	*P* = 0.60
			Knee score Mean (SD) = 86.4 (13.3)	Knee score Mean (SD) = 89.4 (7.5)	Not significant	*P* = 0.35
			Function score Mean (SD) = 87.5 (15.8)	Function score Mean (SD) = 87.3 (19.3)	Not significant	*P* = 0.96
		Revision rate	1 (1.96%)	2 (10%)	Not significant	*P* = 0.13
Baker et al. [28]	2014	OKS	Mean (95% CI)	Mean (95% CI)	Not significant	*P* = 0.56
			Preop = 18.9 (18.7–19.0)	Pre-op = 18.9 (18.8–19.1)		
			Postop = 34.0 (33.8–34.2)	Post-op = 34.0 (33.9–34.2)	Not significant	*P* = 0.96
			Change = 15.2 (14.9–15.4)	Change = 15.1 (15.0–5.3)	Not significant	*P* = 0.69
Garneti et al. [29]	2008	KSS	Total Score Mean (SD) = 161 (33.07)	Total Score Mean (SD) = 156 (52.70)	Not significant	*P* = 0.12
			Knee Score Mean (SD) = 89 (10.62)	Knee Score Mean (SD) = 81 (26.30)	Not significant	*P* = 0.09
			Function Score Mean (SD) = 72 (25.01)	Function Score Mean (SD) = 75 (28.55)	Not significant	*P* = 0.33
		Euroqol score	Mean (SD) = 95 (4.50)	Mean (SD) = 91 (3.76)	Not significant	*P* = 0.26
		Patient-reported Anterior knee pain	5 (7%)	18 (25%)	Not significant	*P* = 0.05
		Patient-reported patellar apprehension	11 (14%)	5 (8%)	Significant	*P* = 0.02*
		Patient-reported knee instability	5 (7%)	4 (6%)	Not significant	*P* = 0.11
		Patient-reported decrease in pain contra-laterally	15 (39%)	10 (27%)	Not significant	*P* = 0.31
		Revision rate	0 (0%)	7 (10%)	Significant	*P* = 0.01*
		Return to preoperative functional level	62 (82%)	58 (88%)	Not significant	*P* > 0.05
		Patient-reported use of walking aid	28 (37%)	37 (41%)	Not significant	*P* = 0.54
		Patient-reported limp	13 (17%)	5 (8%)	Significant	*P* = 0.04*

^*^ = *P* < 0.05, *OKS* Oxford Knee Score, *VAS* Visual Analogue Scale, *TKA* Total knee arthroplasty, *ROM* Range of movement, *KSS* Knee Society Score

## Discussion

There remains a sparsity of literature directly comparing kneeling outcomes between PR and NPR cohorts of patients receiving TKA. Of the studies identified in the systematic search process of this review, only two reported statistically significant findings, with one study favoring PR [26] and another favoring NPR [23].

Wilding et al. reported significantly improved kneeling ability after TKA with PR [26]. Kneeling involves flexion angles of between 120 to 150 degrees, retention of the medial pivot of the medial femoral condyle with posterior movement of the lateral femoral condyle and internal tibial rotation [31]. Early TKA designs neglected the patellofemoral joint (PFJ), and until in 1974, resurfacing of the patella using a polyethylene dome was introduced with the Insall-Burstein total condylar replacement (Zimmer, Warsaw, IN, USA). However, PR presented its own complications, leading to disagreement in the orthopedic community with regards to the optimal intraoperative management of the patella [32]. PR allows for the optimization of modern ‘patellar friendly designs’ by providing an improved congruence between the patella and flange implant surfaces, negating morphological and anatomical variations that may be present in the native patellae. This can improve patellar tracking and overall flexion [33], theoretically positively impacting kneeling ability.

Conversely, Huish et al. reported that NPR patients had a higher self-reported ability to kneel [23]. Resurfacing the patella can also lead to complications that can impact kneeling ability, such as instability, dislocation, aseptic loosening, overstuffing of the PFJ and patellar clunk syndrome [32]. The articulating surface of the patella changes during flexion with patellofemoral pressures peaking at around 90–120 degrees [34] and during high flexion activities, computational and biomechanical studies have demonstrated that the patella undergoes significant sagittal strains that increase inversely to the residual thickness of the patella [35]. Over-resection of the patella during PR can therefore predispose to an increased risk of fracture and pain in deep flexion impacting kneeling ability.

It is important to note that Huish Jr et al. reported kneeling ability as a PROM whereas Wilding et al. additionally assessed kneeling ability clinically. Self-reported kneeling ability has been shown to be inferior to observed kneeling ability [36] and a study comparing TKA, uni-compartmental knee arthroplasty and PFJ replacement reported that, while only 37% of patients thought that they could kneel, 81% were actually able to do so [37]. Palmer et al. found that patients avoided kneeling after TKA for fear of harming the prosthesis and that there were uncertainties regarding the advice given by doctors and nurses [30]. It is uncertain as to why kneeling avoidance advice is given but could be due to concerns regarding wound infection in the early postoperative phase or kinematic concerns regarding increasing patellar loads during high flexion activities, but patient education programs have been shown to improve patient-reported kneeling ability postoperatively [38]. Despite discordance between perceived and actual kneeling ability, six of the eight studies in this review only assessed kneeling as a PROM, with the remaining two also using an additional objective clinical assessment.

Huish Jr et al., however, conducted a follow-up of longer period (both for PR and NPR cohorts) for their study when compared to Wilding et al. This is significant as kneeling PROMs have been shown, in a large prospective study of 5,600 OKS questionnaires, to drastically improve up to a year following surgery and subsequently worsen in the years thereafter, which might be explained by old age of the subjects [39]. In addition, Wilding et al. reported a higher MINORS score when compared to Huish Jr et al., indicating a more robust overall methodological quality (Table 2).

Whether or not to resurface the patella remains a contentious topic, with a lack of clear evidence supporting one technique over the other. A meta-analysis of RCTs in 2005 commented on an increased risk of re-operation with NPR [40] and these findings were echoed in more recent reviews and meta-analyses [41, 42]. In contrast, an RCT comparing PR and NPR in patients with bilateral TKA found no significant difference in revision rates at 10 years [43]. Of the studies included in this review, a study by Garneti et al. reported a significantly increased re-operation rate in the NPR cohort [29], but Huish Jr et al. and Albrecht et al. found no significant difference [23, 27], although it is important to note that, despite absence of statistical significance, both studies reported higher re-operation rates in the NPR group.

Conflicting evidence has also been presented with regards to whether PR influences PROMs. One study found no significant difference in the IKS function score [37] while another reported no short-term differences in terms of KSS scores, but found a significant difference at a longer-term follow-up of five years [41]. Most studies included in this review reported no significant difference with regards to OKS, KSS, Euroqol or Baldini scores. Sangoi et al. did report improved Feller scores for PR, which includes items on anterior knee pain and stair climbing [25] but Garneti et al. found higher rates of patient-reported limp and patellar apprehension in the PR group [29].

Many studies reported no difference between posterior-stabilized (PS) and cruciate-retaining (CR) designs with regards to clinical, functional and radiological outcomes [44, 45]. However, PS knees have been shown to demonstrate higher ROM when compared to CR designs, with some high flexion prostheses having been shown to improve kneeling outcomes [45–47]. Wilding et al. reported that favorable kneeling outcomes remained independent of whether the PCL was retained or sacrificed [26].

Skin incision has been shown to impact kneeling ability, with a recent systematic review and meta-analysis reporting that patients had improved odds of kneeling with anterolateral incisions compared to anteromedial incisions; a transverse incision compared to a longitudinal incision and a shorter incision compared to a longer one [48]. Longer and anteromedial incisions have been reported to result in larger areas of cutaneous sensory change, which can subsequently impact kneeling ability negatively [49, 50]. Two studies in this review used a medial parapatellar approach [25, 30], with one study utilizing a combination of medial parapatellar and subvastus approaches [23] but none of the included studies specifically commented directly on the effect of the type, size or location of the skin incision used.

One of the non-surgical variables associated with kneeling ability is postoperative ROM. Most kneeling postures are high flexion activities with a minimum of 90 degrees of knee flexion required. Wilding et al. reported that knees that were able to flex to at least 100 degrees had significantly improved chances of kneeling [26], which is consistent with studies demonstrating that high flexion TKA designs improved postoperative flexion by 15–25 degrees and also improved the ability to kneel, squat and sit cross-legged [47]. Palmer et al. found the mean ROM was 114 degrees in patients able to kneel and 110 degrees in those unable to do so, but this was not statistically significant [30].

Other non-surgical factors associated with kneeling ability include BMI and gender. An inverse relationship between kneeling ability and BMI has been reported in the literature [51]. This may simply be due to increased amounts of adipose tissue resulting in earlier contact between the posterior thigh and lower leg, thus preventing deep flexion and kneeling, and one study reported that lower BMI was associated with improved kneeling ability in upright and single-leg kneeling positions [52], although another demonstrated no significant relationship between BMI and short-term functional performance following TKA [53]. Rooks et al. reported that patients with a BMI more than 33 were significantly less likely to be able to kneel [24].

Morphological differences between male and female knees previously led to the development of gender-specific implants, but studies looking at whether these differences result in variation in clinical and functional outcomes between men and women have produced conflicting findings [54, 55]. Rooks et al. reported that males were significantly more likely to be able to kneel [24], which was consistent with the findings of a retrospective cohort analysis of 404 patients [51]. Conversely, Huish Jr et al. reported kneeling ability to be higher in females [23]. Age, revision as opposed to primary surgery, and KSS scores were not significantly associated with kneeling ability post-TKA [24, 26, 30].

### Limitations

Kneeling and the ability to kneel are poorly defined and infrequently reported in the literature, limiting the number of studies appropriate for inclusion. Variability also remains in how this outcome is assessed, often using Question 7 of the OKS or Question SP5 of the KOOS. There are, however, issues, because Question 7 of the OKS is not designed to be used in isolation to assess kneeling ability and neither question considers kneeling position, kneeling duration, the surface knelt upon, social, cultural or occupational importance. There is also variability with regards to how kneeling is assessed, which makes comparisons difficult. Due to the heterogeneous nature of the data, it was not possible to perform a meta-analysis.

Comparing PR and NPR is difficult because there is a lack of standardization in the reporting of the surgical techniques and designs of implants used. There are also various options available to the surgeon with regards to the intraoperative management of the patella, including circumpatellar dennervation, osteophyte removal, patelloplasty and lateral retinacular release, which can be performed in isolation or combination, but once again, but these were not clearly reported.

Kneeling is a complex multi-joint movement and hence also is affected by global disease, with arthritis of the spine, hips or contralateral knee affecting kneeling ability regardless of the postoperative outcomes of a TKA.

## Conclusions

Despite being highly important to patients and known to impact patient satisfaction, kneeling remains ill-defined, under-reported and under-investigated as an outcome measure. The available data regarding intra-operative variables such as PR and its impact on postoperative kneeling ability consists largely of small retrospective studies with no randomized studies identified in the systematic search of this review process. The results were conflicting, with a statistically significant association being identified in only two studies, one favoring PR and the other NPR. Other factors associated significantly with kneeling included BMI, postoperative flexion, and gender, with one study favoring females and the other males. No significant differences were observed between PR and NPR with regards to OKS, KSS and Euroqol scores. However, an increased rate of re-operation was reported in the NPR group. In the absence of large, randomized data sets comparing kneeling ability in PR and NPR patients, it is difficult to reach meaningful conclusions. In order to clarify the situation, large, long-term prospective randomized controlled studies (RCTs) are required with clear definitions of what kneeling entails and how to assess it best, so that standardized reporting can be performed and compared.

## Data Availability

Not applicable.

## Abbreviations

CR: Cruciate-retaining
CS: Cruciate-sacrificing
HDAS: Healthcare database advanced search
IKS: International knee society
KOOS: Knee injury and osteoarthritis outcome score
KSS: Knee society score
MeSH: Medical subject heading
MINORS: Methodological index for non-randomised studies
NJR: National joint registry
NPR: Non-patellar resurfacing
OA: Osteoarthritis
OCEBM: Oxford centre for evidence-based medicine
OKS: Oxford knee score
PCL: Posterior cruciate ligament
PFJ: Patellofemoral joint
PR: Patellar resurfacing
PRISMA: Preferred reporting items for systematic reviews and meta-analyses
PROM: Patient-reported outcome measure
PROSPERO: International prospective register of systematic reviews
RCT: Randomized control trial
ROM: Range of movement
TKA: Total knee arthroplasty
